# Supplementary material for: Dysfunction of duplicated pair rice histone acetyltransferases causes segregation distortion and an interspecific reproductive barrier
Source: Nat Commun. 2024 Feb 2;15:996. doi: 10.1038/s41467-024-45377-x (PMC10837208; doi:10.1038/s41467-024-45377-x)
Supplement: Supplementary file 1 — Supplementary Information [file 41467_2024_45377_MOESM1_ESM.pdf]

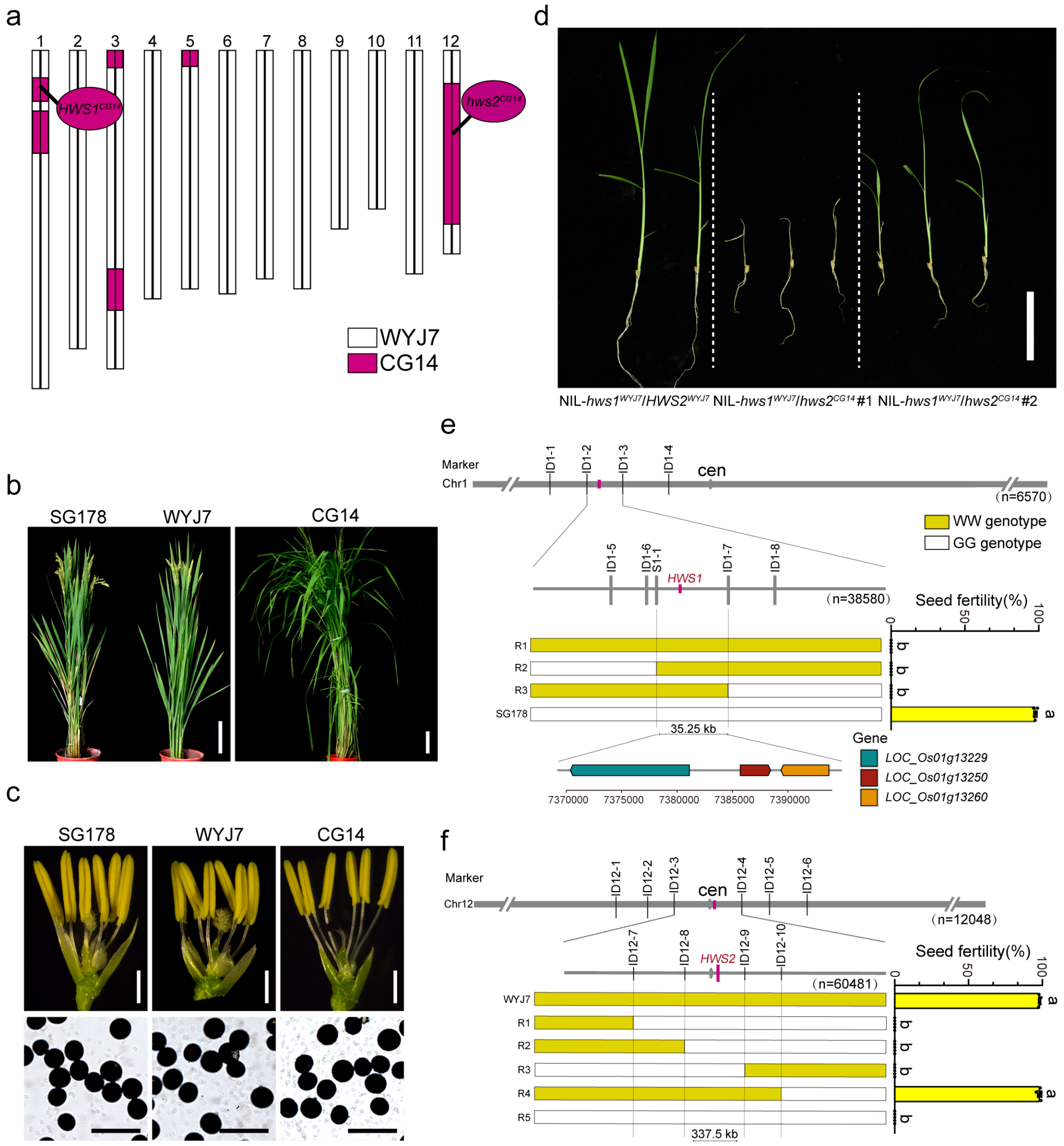

Supplementary Fig. 1 Map-based cloning of *HWS1* and *HWS2*. a, Graphical genotype of SG178. The Affymetrix SNP chip was used in high-throughput SNP genotyping for genetic investigation. The two ovals indicate the gene locations of *HWS1* and *HWS2*, and the pink regions represent the chromosomal substitution segments derived from the donor variety CG14. **b**, Morphological comparison of mature plants of SG178 and the parental lines WYJ7 and CG14. Scale bars = 15 cm (left) and 10 cm (right). **c**, Fertility investigation of the rice lines in **b**. Morphologies of mature spikelets (top, 2 mm) and microscopic observation of stained pollen grains (bottom, 100 μm) are shown. **d**, The lethal phenotype (#1) and growth arrest (#2) in germinating self-pollinated progeny plants from *HWS2* hybrids at the seedling stage. Scale bar = 3 cm. **e**, **f**, Fine mapping of *HWS1* (**e**) and *HWS2* (**f**). The number of

identified plants, genetic markers, and high-resolution linkage maps are shown. The genotypes and relative seed fertility of key recombinant individuals are shown in the bottom right section. All candidate genes predicted in the *HWS1* target region are given in (**e**). The yellow and white bars indicate the homozygous WYJ7 (WW) and CG14 (GG) genotypes, respectively. Data are mean ± SEM (n= 5 plants). Different letters denote significant differences (p <0.05, one-way ANOVA with two-sided Tukey's HSD test). P values for **e** and **f** are adjusted and shown in the **Source Data** file.

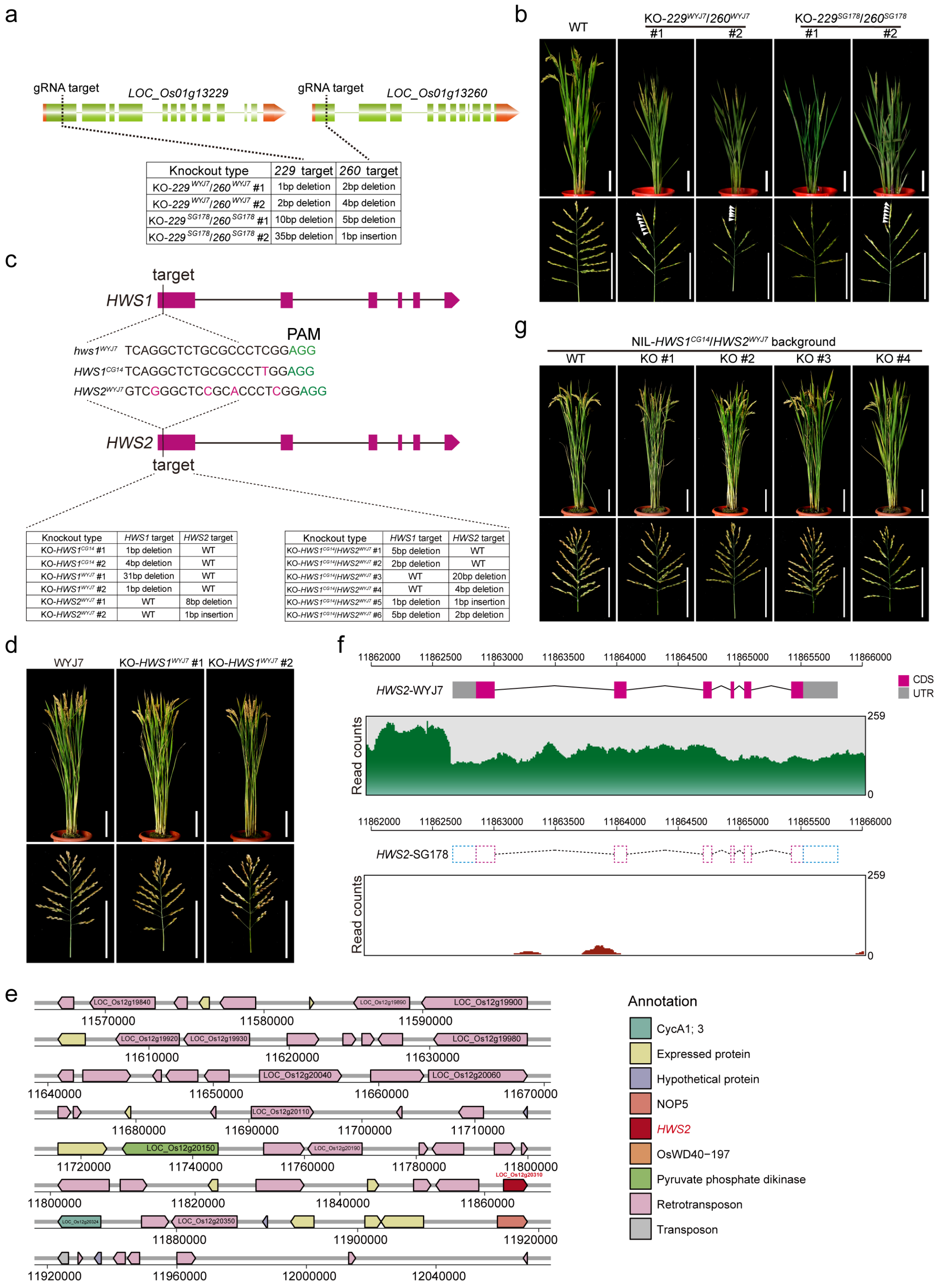

Supplementary Fig. 2 Transgenic validation of the *HWS1/2* candidate genes and genome structure of the *HWS2* locus determined by DNA resequencing. a, The edited sequences of *229*/*260* in the CRISPR-Cas9 knockout (KO) lines. Gene structures and the locations of the gRNA targets are shown. **b**, Primary observation to T2-generation *229*/*260* double-mutant plants in the WYJ7 and SG178 backgrounds. Full

seeds are indicated with white arrowheads. Scale bars = 10 cm. **c**, Generation and characterization of *HWS1/2* loss-of-function mutants using CRISPR/Cas9-mediated gene editing and DNA sequencing. Gene structures are shown for *HWS1* and *HWS2*. The pink boxes indicate the exons and the black lines indicate the introns. The positions of the target sites are indicated with vertical lines and the unmutated sequences of $hws1^{WYJ7}$, $HWS1^{CG14}$, and $HWS2^{WYJ7}$ are shown. The different mutant alleles of *HWS1* and *HWS2* are given in the tables below the figure. **d**, Phenotypes of mature single *HWS1* KO plants and panicles edited in WYJ7 background. Scale bars = 15 cm (top) and 10 cm (bottom). **e**, Predicted candidate genes are depicted by colored boxes with respective gene names in the 662-kb chromosomal region containing the *HWS2* locus according to the MSU Rice Genome Annotation Project Database (http://rice.uga.edu). **f**, Genome browser views of read coverage across the *HWS2* genomic region in WYJ7 and SG178 based on next-generation sequencing data. The number of mapped reads is shown, and very few reads were detected for the *HWS2* in SG178. **g**, Phenotypes of mature single *HWS1* or *HWS2* KO plants and panicles edited in the NIL-$HWS1^{CG14}$/$HWS2^{WYJ7}$ background. Scale bars = 15 cm (top) and 10 cm (bottom).

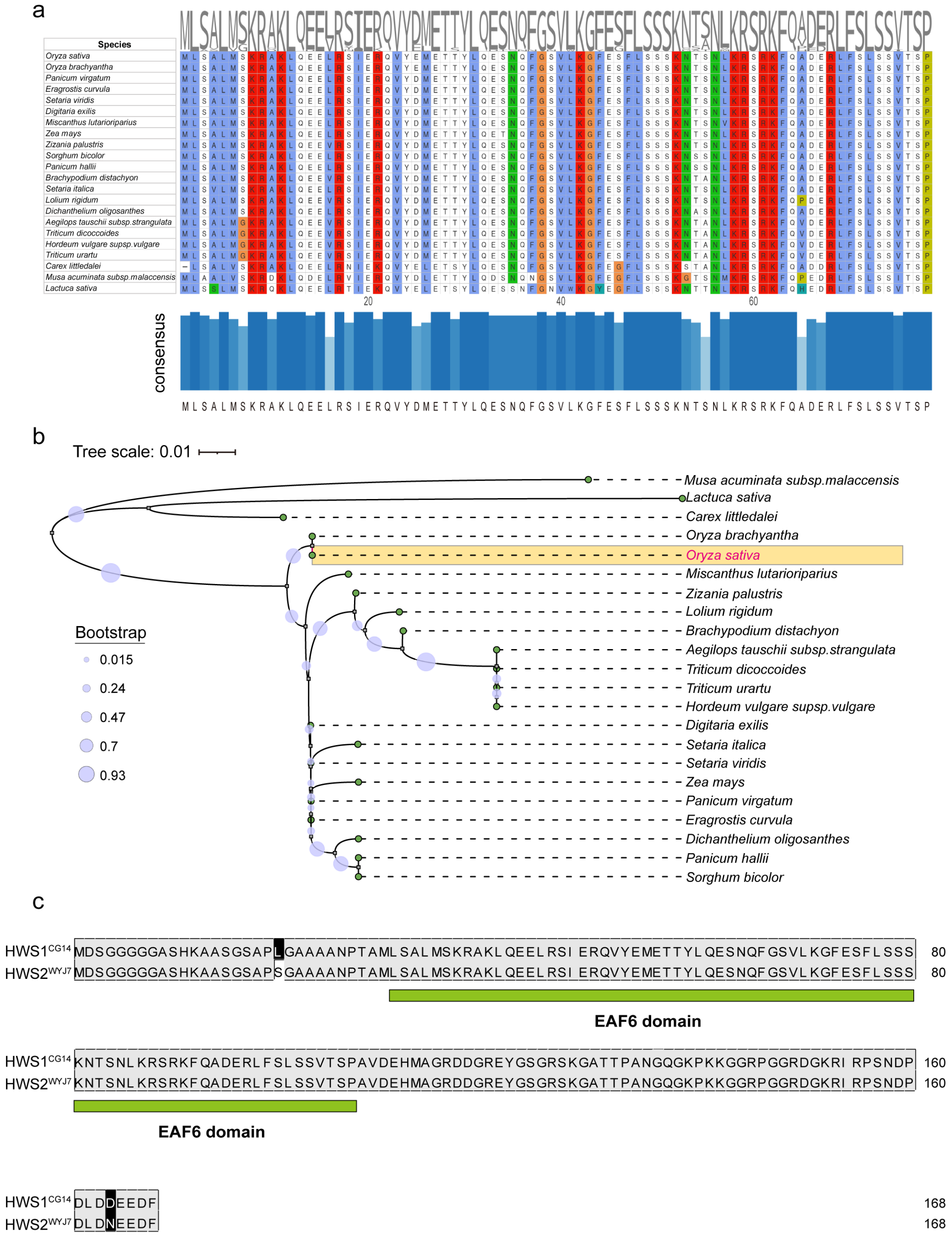

Supplementary Fig. 3 Phylogenetic analysis and sequence comparison of HWS1/2 and their homologs. a, Analysis of conserved amino acids in homologous EAF6 domains of the HWS1/2 protein orthologs in representative monocot and dicot species. **b**, Neighbor-joining phylogenetic tree for HWS1/2 and their homologs was constructed using MEGA 11.0 software with default parameters based on the EAF6 domain sequence. The relative bootstrap values derived from 1,000 bootstrap replicates are shown as lavender circles on the branches. **c**, Amino acid sequence alignment of HWS1

from CG14 and HWS2 from WYJ7. The amino acid substitutions are shaded in black. The predicted EAF6 domain (amino acids 30-107) is indicated by the labeled green bar.

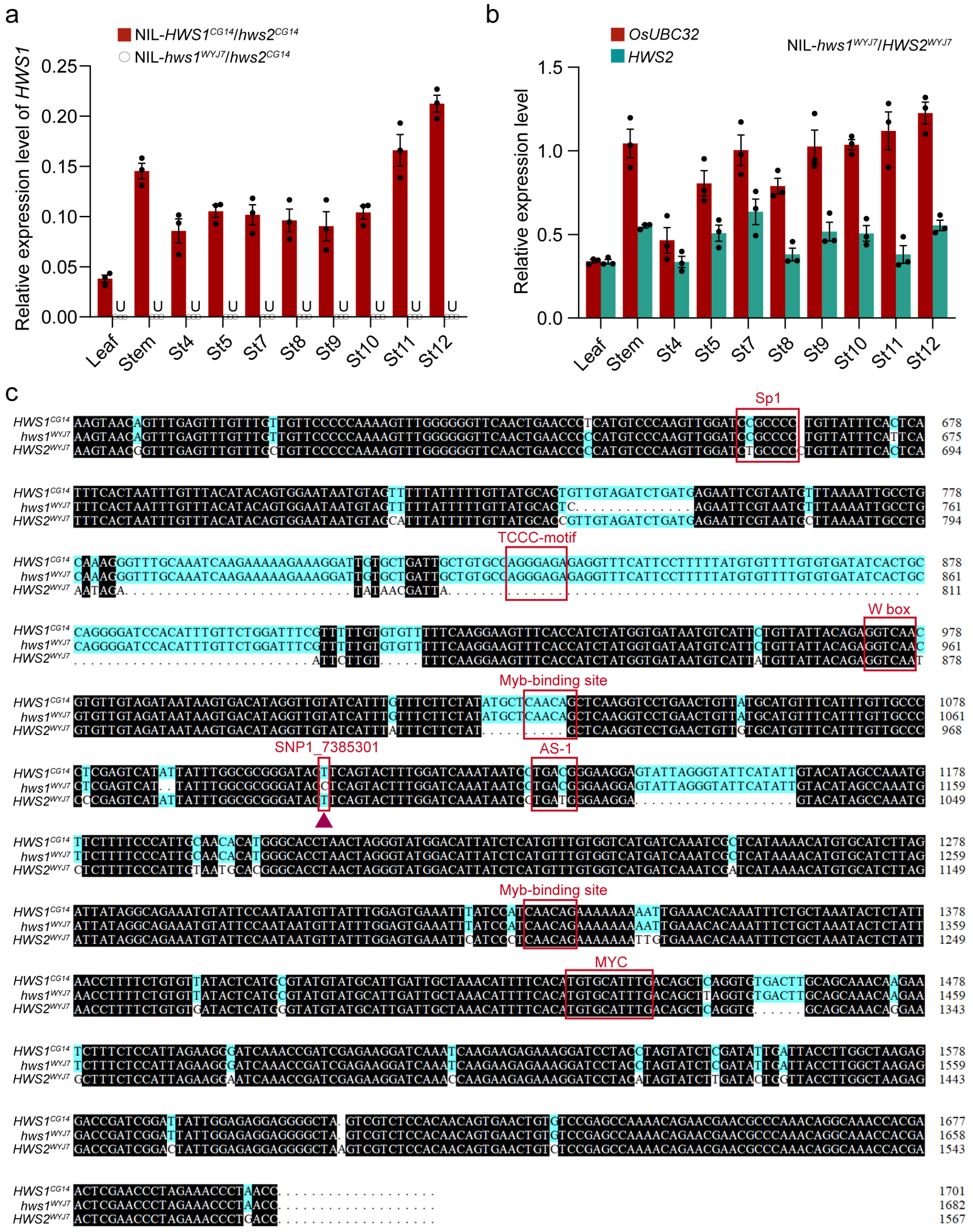

Supplementary Fig. 4 Feature analysis of the *HWS1* and *HWS2* genes. a, **b**, Expression pattern analysis of *HWS1* (**a**) and *HWS2* (**b**). St4 to St12 indicate the different anther developmental stages. U, undetectable expression. Quantification of *OsUBC32* gene expression was used as the reference in comparison to *HWS2* gene expression levels in NIL-*hws1*^{WYJ7}/*HWS2*^{WYJ7} plants. Data are shown as mean ± SEM (n = 3 biological replicates). Source data are provided in the **Source data** file. **c**, Sequence analysis of the putative promoter regions of *HWS1* and *HWS2* using

DNAman software. The boxed SNP indicated by a red arrowhead is predicted to be highly associated with gene expression level. The predicted *cis*-acting regulatory elements retrieved from the PlantCARE (https://bioinformatics.psb.ugent.be/webtools/plantcare/html/) database in promoter are framed in red.

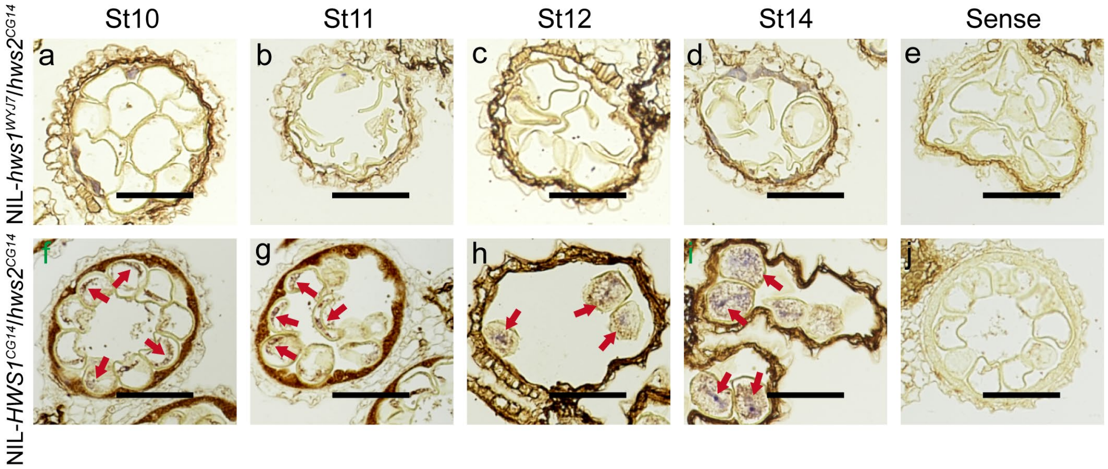

Supplementary Fig. 5 Expression pattern analysis of *HWS1* in the pollen grain. **a-j**, RNA in situ hybridization analysis of *HWS1* in anther at early pollen development stages. Transverse sections of NIL-$hws1^{WYJ7}/hws2^{CG14}$ (**a-e**) and NIL-$HWS1^{CG14}/hws2^{CG14}$ (**f-j**) developing anthers were hybridized with $HWS1^{CG14}$ antisense (**a-d** and **f-i**) or sense probes (**e** and **j**). Red arrows showed expression signals of *HWS1* in the pollen grains. Scare bar = 50 μm. All experiments were repeated independently at least three times of at least three plants, with similar results.

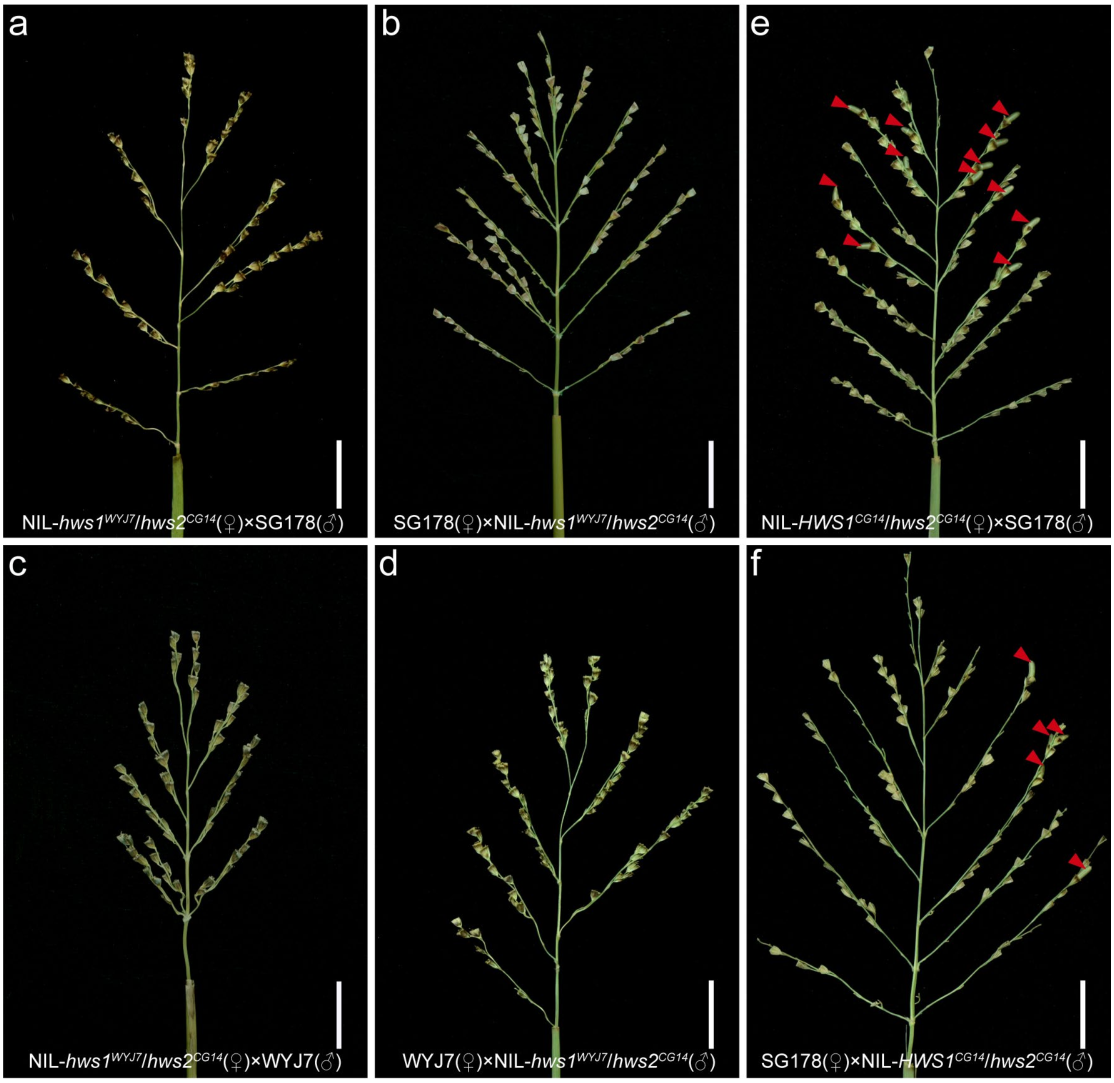

Supplementary Fig. 6 Investigation of reciprocal cross effects in NIL-*hws1*^{WYJ7}/*hws2*^{CG14}. a-f, Reciprocal cross experiments showing the sterility character in panicles of NIL-*hws1*^{WYJ7}/*hws2*^{CG14} plants (**a-d**) compared with NIL-*HWS1*^{CG14}/*hws2*^{CG14} panicles (control, **e**, **f**). SG178 and WYJ7 were used as the maternal or paternal parents in the crosses. The details of each cross are shown at the bottom of each panel. Red arrowheads indicate filled seeds after pollination. Scale bars = 10 cm.

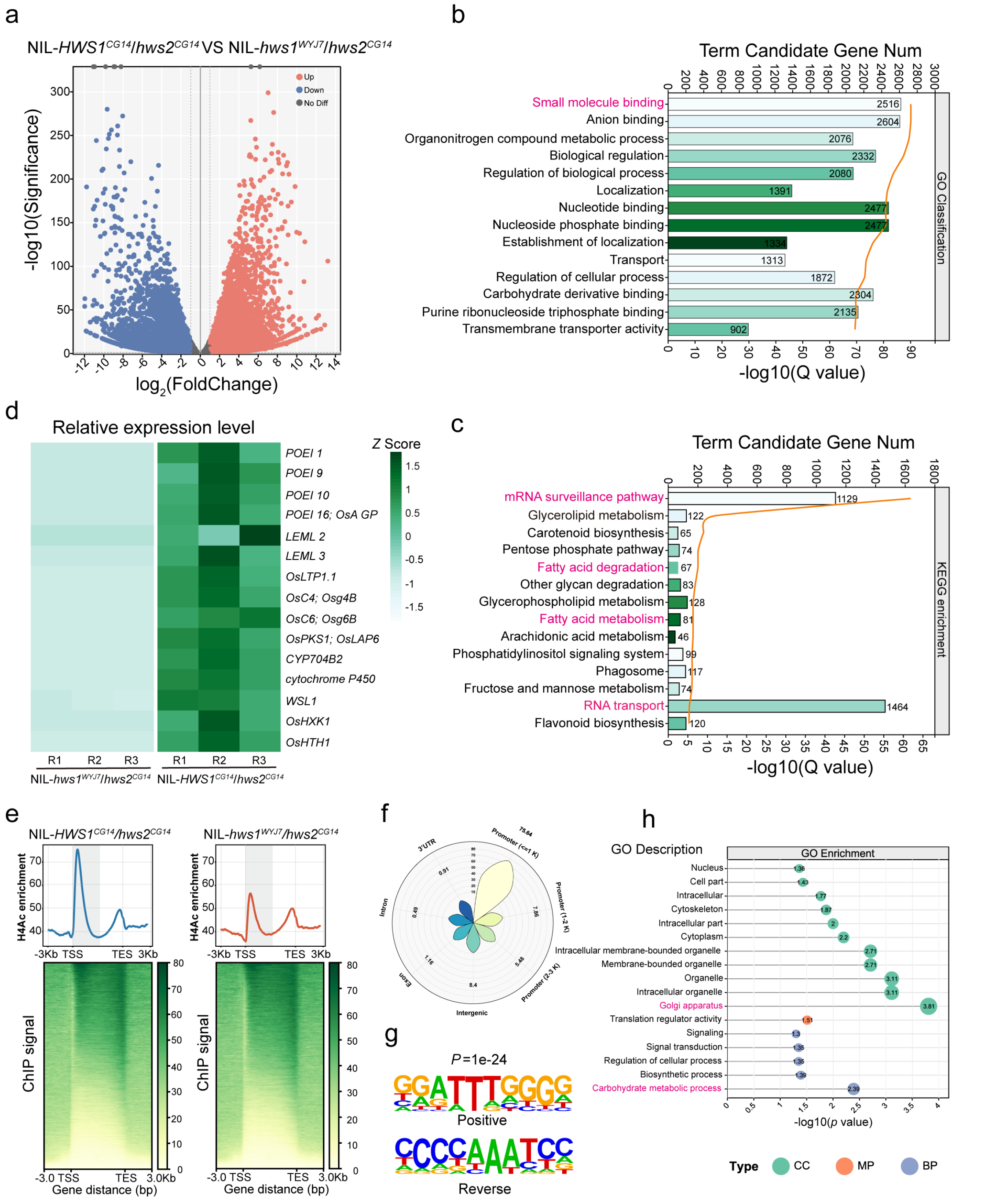

Supplementary Fig. 7 The combined analysis of transcriptomic and epigenomic data. a, Volcano plot of the differentially-expressed genes (DEGs) between NIL-*HWS1*^{CG14}/*hws2*^{CG14} and NIL-*hws1*^{WYJ7}/*hws2*^{CG14}. Red, blue, and grey dots represent genes with higher, lower, or unchanged expression, respectively. **b**, **c**, GO enrichment (**b**) and KEGG pathways analysis (**c**) of DEGs from (**a**). The top 20 GO and KEGG categories in the DEGs are displayed on the left side of each graph. Yellow lines indicate the Q values (adjusted *p* value, n = 3 samples), and green rectangles show the number of candidate genes in each designated term. The *p* values were calculated based

on the hypergeometric distribution model and were adjusted to Q values using the Benjamini-Hochberg method. **d**, Relative transcript levels of a series of downregulated genes in NIL-*hws1*^{WYJ7}/*hws2*^{CG14} that were previously reported or annotated to cause male or female sterility, as shown by a heat map. p <0.05, n = 3 samples. **e**, Average density plot (upper panels) and heatmap (lower panels) showing genome-wide histone H4 acetylation profiles from TSS to TES in NIL-*HWS1*^{CG14}/*hws2*^{CG14} and NIL-*hws1*^{WYJ7}/*hws2*^{CG14} plants. Each row represents the normalized acetylated histone H4 level over a gene, and the gradient colors present the enrichment level. **f**, Rose map showing the distribution and composition of differentially enriched peaks across the rice genome. **g**, Motif analysis using HOMER to identify core motifs enriched in the peaks. **h**, GO analysis of the overlapping genes (downregulated genes with weakened ChIP-seq signals) in NIL-*hws1*^{WYJ7}/*hws2*^{CG14}. Significantly enriched GO categories were identified using ClusterProfiler (identification) and FDR (*p*-values correction). Major GO categories CC, Cellular Component; MF, Molecular Function; BP, Biological Process.

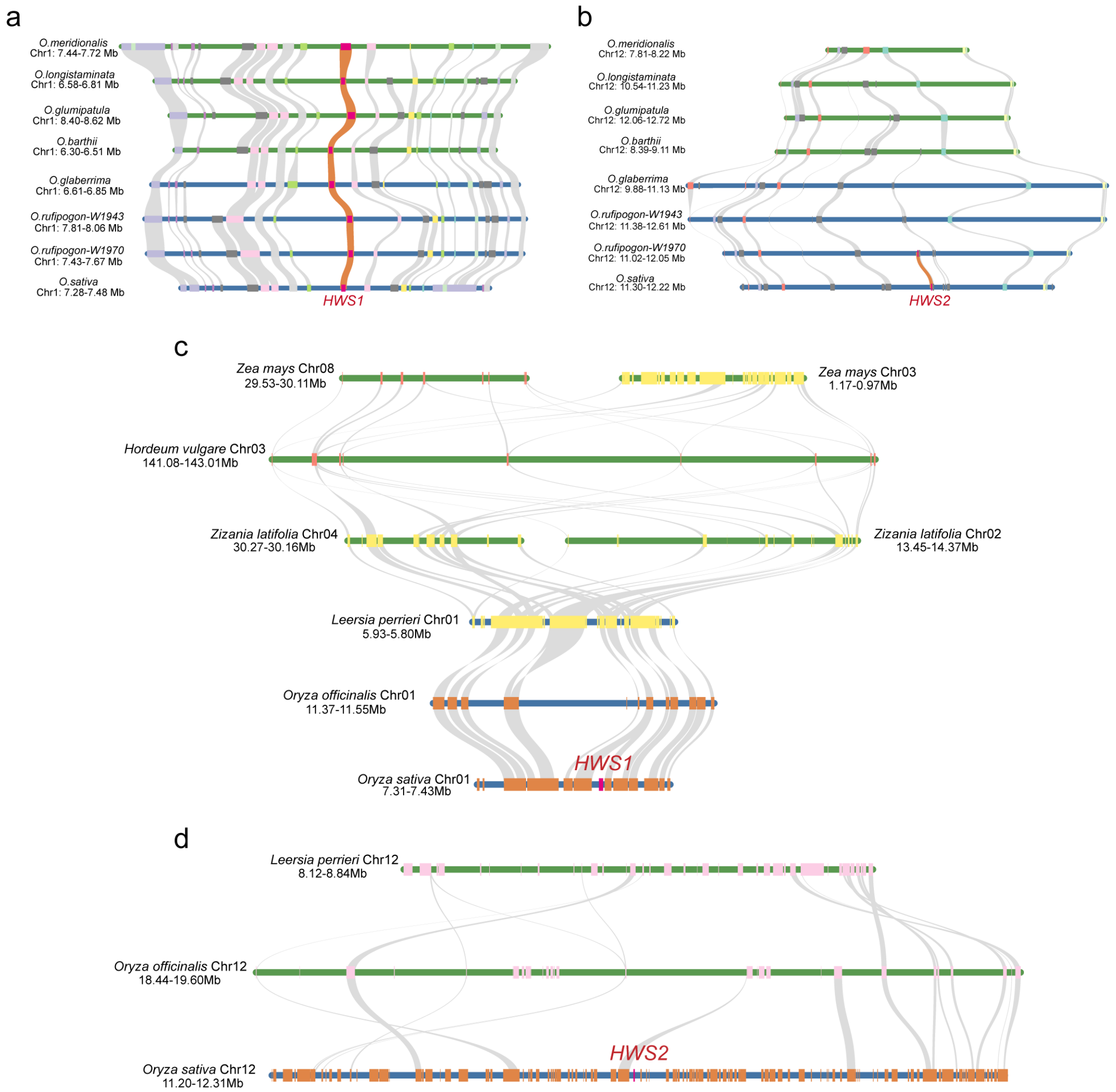

Supplementary Fig. 8 Local synteny analysis of the *HWS* loci in rice. a, **b**, Gene synteny analysis of *HWS1* (**a**) and *HWS2* (**b**) between *O.sativa*, *O.rufipogon*, *O.glaberrima*, *O.barthii*, *O.glumipatula*, *O.longistaminata*, and *O.meridionalis*. In **a** and **b**, orange lines indicate conserved syntenic blocks harboring *EAF6* (*HWS1/2*). **c**, Local synteny analysis of *HWS1* between *O.sativa* and the other five grasses. **d**, Local synteny analysis of *HWS2* between *O.sativa*, *O.officinalis*, and *L.perrieri*. In **a-d**, conserved syntenic blocks are connected by grey (homologous genomic regions), and the locations of *HWS1/2* and their orthologs in each species are shown in pink.

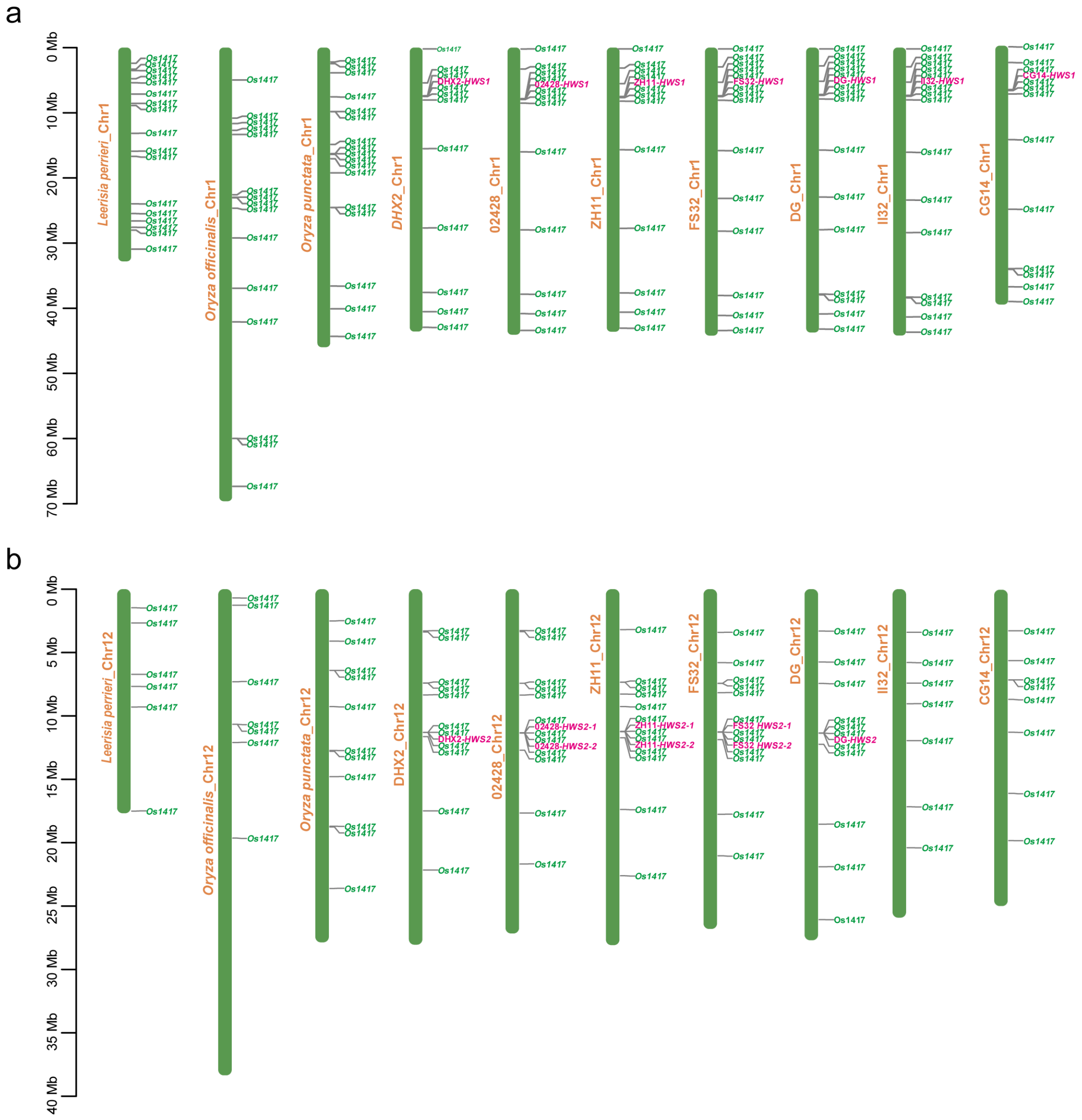

Supplementary Fig. 9 Transposon analysis of the *HWS* loci in rice. a, **b**, The insertion sites of DNA transposons (*Os1417*) labelled in green are shown along the chromosomes in *L. perrieri* and *Oryza* species/cultivars. The *HWS1* (**a**) and *HWS2* (**b**) loci are highlighted in pink on each chromosomal diagram. The species and cultivar names are shown yellow on the left of the long rectangles that represent the chromosomes. The analyzed species include *L. perrieri* (putative diploid ancestor of rice), *O. officinalis* (CC-genome rice species), *O. punctata* (BB-genome rice species), and 7 AA-genome rice cultivars consisting of *O. sativa japonica*, *indica* and *O. glaberrima* groups (n=3, 3, and 1, respectively).

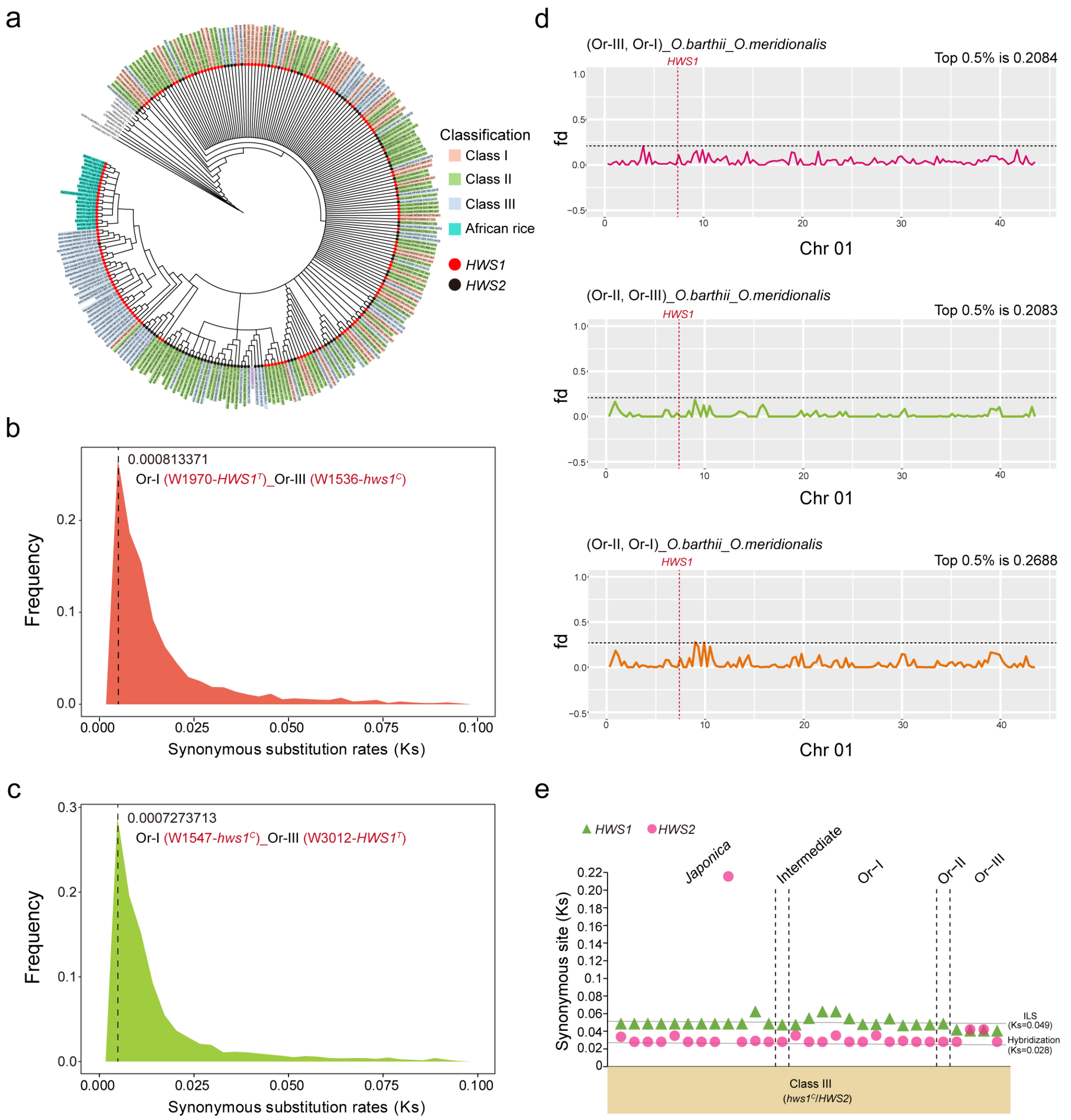

Supplementary Fig. 10 Distinguish between ILS and introgression event. a, A phylogenetic tree was constructed from an alignment of the complete coding sequences of the *EAF6* genes (*HWS1* and *HWS2*) to investigate their evolutionary relationships. *Z. mays*, *H. vulgare*, *Z. latifolia*, *L. perrieri*, *O. officinalis*, *O. punctata*, and 133 AA-genome rice accessions were included in the analysis. *HWS1* and *HWS2* are marked with red and black dots, respectively. The names of each species and rice variety are given and shaded in different colors in the external layer of the ring (Class I, pale pink; Class II, light green; Class III, baby blue; African rice, blue-green). The clade of *HWS1* genes from African rice is closer to that of *hws1*^{C} from the Class III varieties. **b**, **c**, Ks distributions for species between Or-I (W1970) and Or-III (W1536) (**b**), as well as between Or-I (W1547) and Or-III (W3012) (**c**). The dotted lines and numbers indicate the peak frequency of Ks. **d**, Introgression fd distributions of ABBA-BABA test on

chromosome 1 in each of the three paired-topology categories (Or-III, Or-I)_*O.barthii_O.meridionalis* (red line or top panel), (Or-II, Or-III)_*O.barthii_O.meridionalis*, (green line or middle panel), and (Or-II, Or-I)_*O.barthii_O.meridionalis* (orange line or bottom panel). The *HWS1* locus in each paired-topology category is indicated with the red dotted line and the horizontal dotted lines in each paired-topology category indicate the top 0.5% fd value. **e**, Ks distribution of *HWS1/2* in Classes III of the 133 selected Asian accessions or cultivars relative to the *HWS1* of *O.meridionalis*. The taxonomic groups are demarcated by dashed lines, the duplication and ILS event were labelled in the right panel.

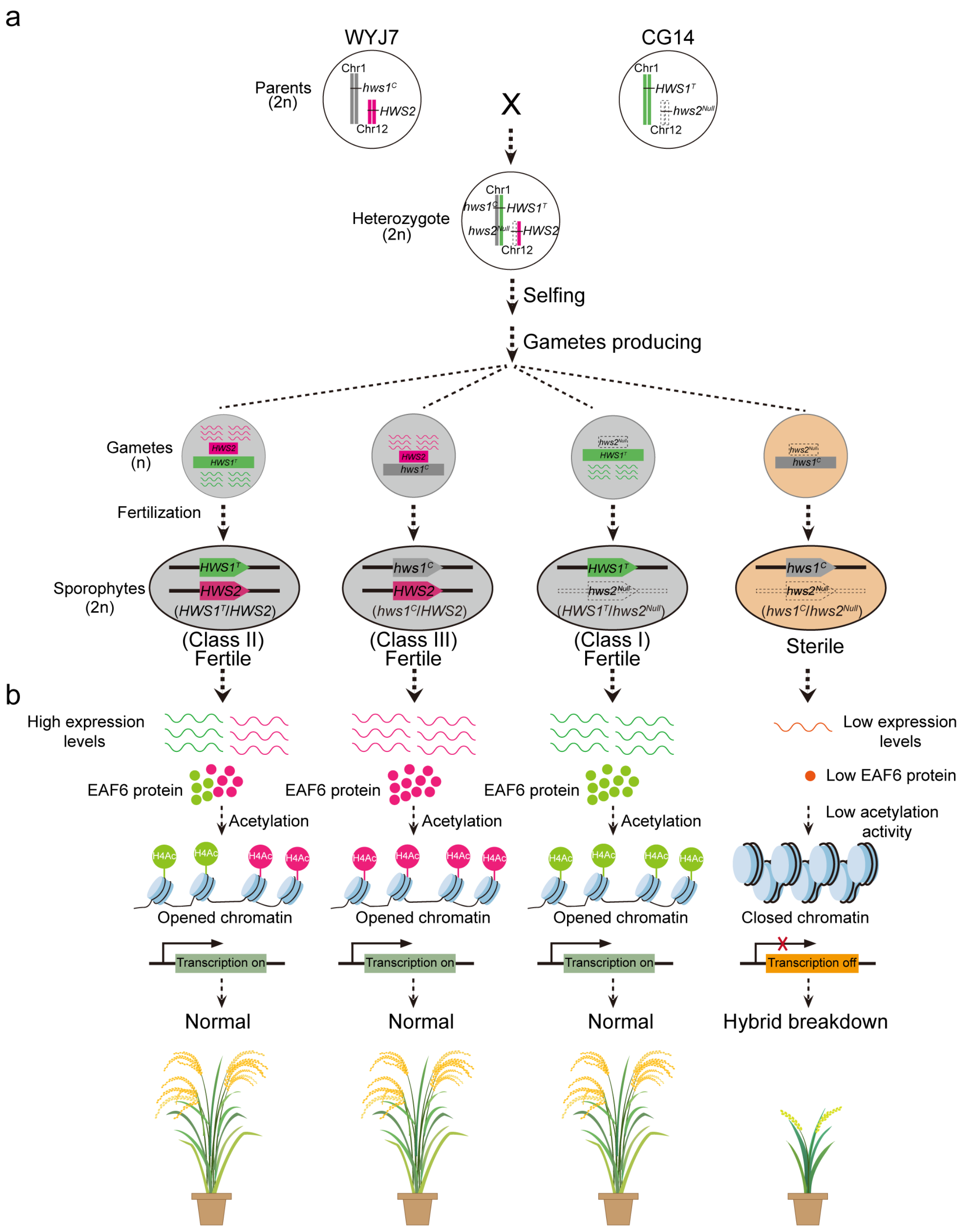

Supplementary Fig. 11 A proposed functional model showing the sterility mechanism via epigenetic modification. a, The duplicated-gene recessive lethal model to explain the genetic nature of Class I-III and completely sterility individuals in the F_2 generation of WYJ7 and CG14 cross combination. Postzygotic reproductive isolation occurs due to the sterility of the hybrid offspring derived from an African-Asian rice cross. In the *japonica* population, one member of a duplicated *EAF6* gene pair (*hws1*^{C}) does not work due to expression failure while the other copy (*HWS2*)

retains its function. In African rice, one *EAF6* member (*$HWS1^T$*) is functional whereas the second (*$hws2^{Null}$*) is completely missing. Individual plants with the *$hws1^C$/$hws2^{Null}$* allelic combination from the F_2 population by selfing reduce the fitness and produce abortive male and female gametes, which gives rise to complete sterility, which reduces the fitness of the hybrids. The colored columns inside the circles indicate the chromosomes. The variations and their combinations are defined in **Figure. 5K**. **b**, A working model showing the mode of HWS1/2 action as a hub that regulates transcription via epigenetic modification. In the F_2 homozygous individuals lacking both HWS1 and HWS2, transcriptional-repression is triggered along with the loss of H4Ac, contributing to reduced expression of *HWS1/2* downstream regulators that are critical for reproduction and vegetative growth. The dots (green or pink, high abundance; orange, low abundance) symbolize the EAF6 protein. The acetylated chromatin is labelled with red circles, and the resulting transcriptional status is described. In **a** and **b**, the wavy lines (green or pink, high expression; orange, low expression) symbolize the *EAF6*-specific mRNA, and the dashed lines represent the loss of the *HWS2* locus.

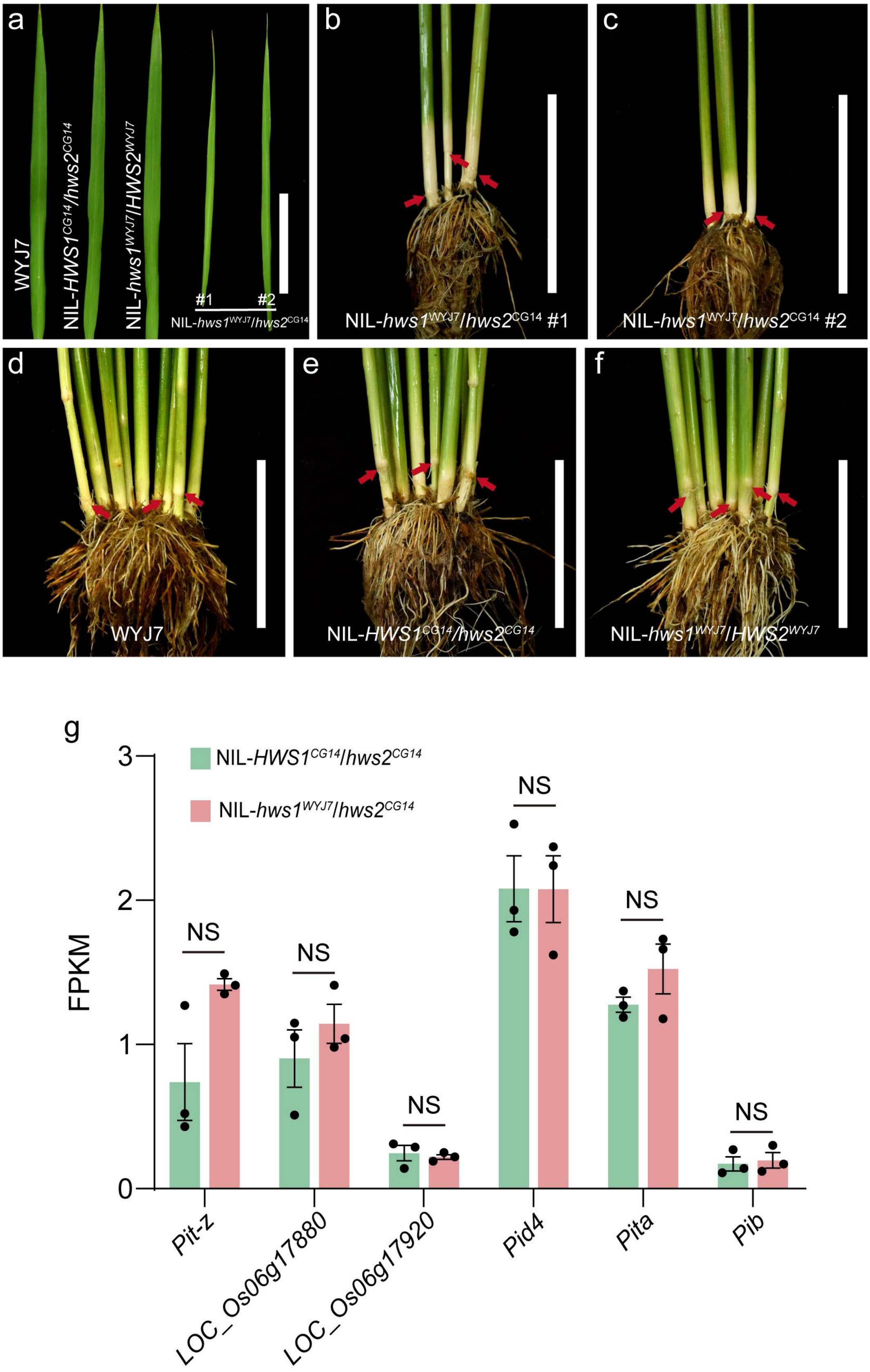

Supplementary Fig. 12 Exclusion of autoimmune response pathway and expression level of six *R* genes. a-f, Phenotype of leaves (**a**) and basal nodes (**b-f**) among NIL-*hws1*^{WYJ7}/*hws2*^{CG14}, WYJ7, NIL-*HWS1*^{CG14}/*hws2*^{CG14}, and NIL-

hws1^{WYJ7}/*HWS2*^{WYJ7} plants. The red arrows indicate the normal basal nodes. Scale bars = 10 cm. **g**, Expression level of six *R* genes in NIL-*hws1*^{WYJ7}/*hws2*^{CG14} versus NIL-*HWS1*^{CG14}/*hws2*^{CG14} plants. FPKM, Fragments Per Kilobase per Million mapped reads. Data are mean ± SEM (n = 3 biological replicates). Significant differences were determined by a two-tailed Student's paired *t*-test. NS, no significance. Source data underlying the statistical analysis are provided in the **Source data** file.

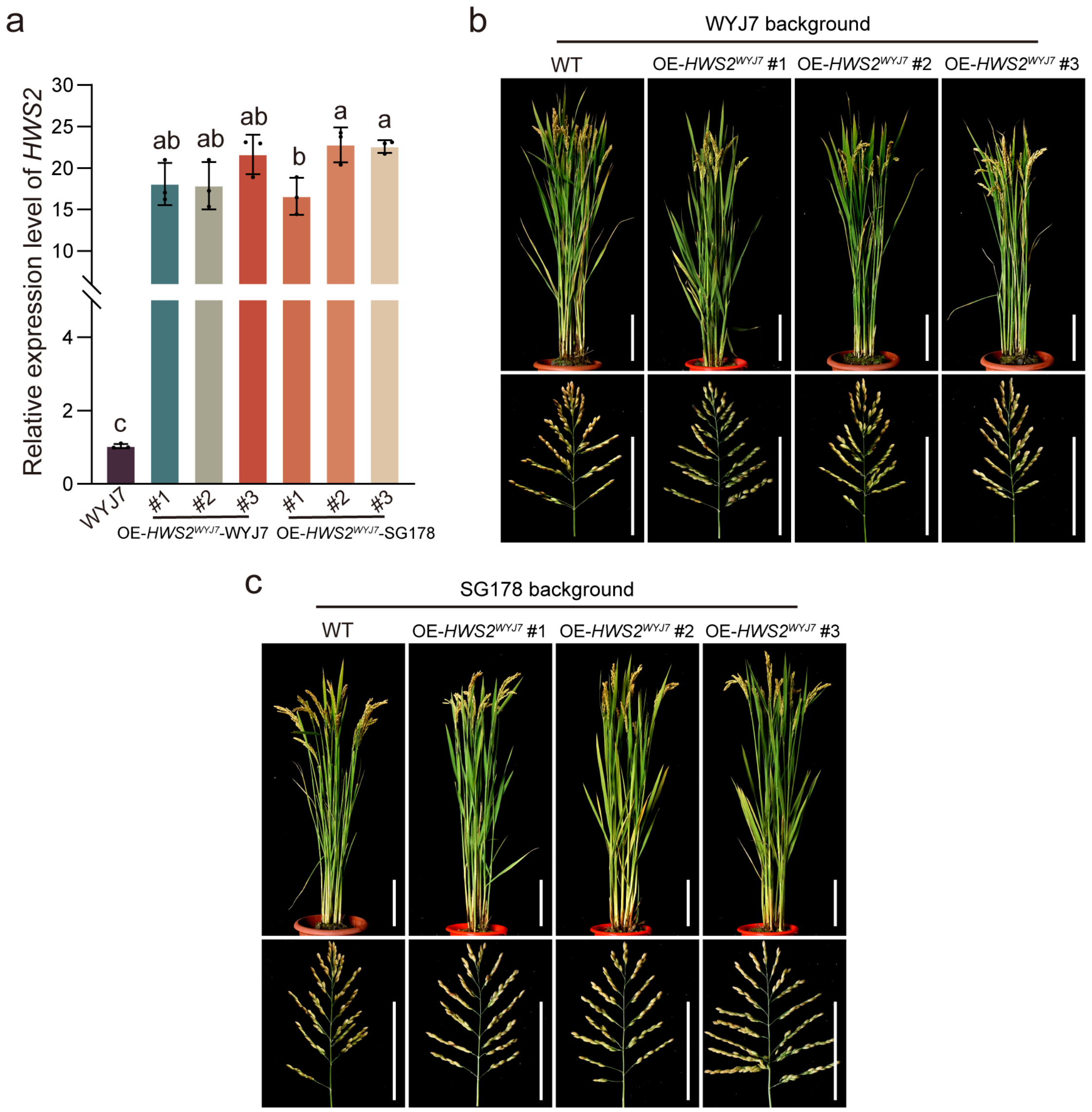

Supplementary Fig. 13 Phenotypic assessment of *HWS2*-overexpressing transgenic plants. a, Relative expression levels of *HWS2* in three independent transgenic overexpression (OE) lines in the WYJ7 and SG178 backgrounds (n = 3 biological replicates). The rice *Ubiquitin* gene was used for normalization of gene expression. Data are mean ± SEM (n=3 biological replications). Different letters denote significant differences (p <0.05, one-way ANOVA with two-sided Tukey's HSD test). P values are adjusted and shown in the **Source Data** file. **b**, **c**, Phenotypes of *HWS2* OE line plants in the WYJ7 (**b**) and SG178 (**c**) backgrounds at maturity. Scale bars = 15 cm (top) and 10 cm (bottom).

Supplementary Table 1. Chi-square test of observed ratio of genotypes in the progenies of the hybrid NIL-*HWS1*/*HWS2*.

Genotype		%G2G2	NO. of individuals		χ^2(1:2:1)	Phenotype
*HWS1*_M1	*HWS2*_K1	plant	Expected	Observe		
W1W1	W2W2		70.5	99(7)		F
	G2G2	6.19	70.5	14(1)		S
	W2G2		141	113(8)		F
G1G1	W2W2		70.5	82		F
	G2G2	20.70	70.5	82	81.864***	F
	W2G2		141	168		F
W1G1	W2W2		141	172		F
	G2G2	24.70	141	118		F
	W2G2		282	280		F
Total			1128	1128		
				n=9		
				df=8		
			$\chi^2_{0.05}(8) = 15.51$ $\chi^2_{0.01}(8) = 20.09$			

W1W1, W1G1, and G1G1 indicate WYJ7 homozygous, heterozygous, and CG14 homozygous in *HWS1* locus, respectively.

W2W2, W2G2, and G2G2 indicate WYJ7 homozygous, heterozygous, and CG14 homozygous in *HWS2* locus, respectively.

The observed segregation ratio of the genotype fit the expected ratio at the 5% significance level except the W1W1|G2G2 genotype (marked red).

***p = 2.06E-14 <0.001 in the two-sided χ^2 test.

Supplementary Table 2. Allele frequency of *hws1*^{C} and *HWS1*^{T} in *Japonica* and *Glaberrima* populations.

Populations	Population size	Frequency of *hws1*^{C} allele	Frequency of *HWS1*^{T} allele	Not detected
Japonica	1512	96.80%	0.03%	3.17%
Glaberrima	103	0%	85.40%	14.60%

The allele frequency of *HWS1*^{T} and *hws1*^{C} from 1512 *Japonica* populations was obtained from the public rice MBKBASE database (https://mbkbase.org/rice).

The allele frequency of *HWS1*^{T} and *hws1*^{C} from 103 *Glaberrima* populations was collected from previously published study[52].
