# Supplementary material for: Dysfunction of duplicated pair rice histone acetyltransferases causes segregation distortion and an interspecific reproductive barrier
Source: Nat Commun. 2024 Feb 2;15:996. doi: 10.1038/s41467-024-45377-x (PMC10837208; doi:10.1038/s41467-024-45377-x)
Supplement: Supplementary file 3 — Description of Additional Supplementary Files [file 41467_2024_45377_MOESM3_ESM.pdf]

Description of additional Supplementary files

File Name: Supplementary Data 1

Description: All accessions used for the evolution and duplication analysis

File Name: Supplementary Data 2

Description: Accessions for the functional SNP analysis

File Name: Supplementary Data 3

Description: All the identified SNPs by using the 41 accessions

File Name: Supplementary Data 4

Description: All the primers used in this study
