# Supplementary material for: Dysfunction of duplicated pair rice histone acetyltransferases causes segregation distortion and an interspecific reproductive barrier
Source: Nat Commun. 2024 Feb 2;15:996. doi: 10.1038/s41467-024-45377-x (PMC10837208; doi:10.1038/s41467-024-45377-x)
Supplement: Supplementary file 8 — Reporting Summary [file 41467_2024_45377_MOESM8_ESM.pdf]

nature portfolio

Corresponding author(s): Hong-Xuan Lin

Last updated by author(s): Jan 8, 2024

Reporting Summary

Nature Portfolio wishes to improve the reproducibility of the work that we publish. This form provides structure for consistency and transparency in reporting. For further information on Nature Portfolio policies, see our Editorial Policies and the Editorial Policy Checklist.

Statistics

For all statistical analyses, confirm that the following items are present in the figure legend, table legend, main text, or Methods section.

n/a	Confirmed	
☐	☒	The exact sample size (n) for each experimental group/condition, given as a discrete number and unit of measurement
☐	☒	A statement on whether measurements were taken from distinct samples or whether the same sample was measured repeatedly
☐	☒	The statistical test(s) used AND whether they are one- or two-sided<br>*Only common tests should be described solely by name; describe more complex techniques in the Methods section.*
☒	☐	A description of all covariates tested
☐	☒	A description of any assumptions or corrections, such as tests of normality and adjustment for multiple comparisons
☐	☒	A full description of the statistical parameters including central tendency (e.g. means) or other basic estimates (e.g. regression coefficient) AND variation (e.g. standard deviation) or associated estimates of uncertainty (e.g. confidence intervals)
☐	☒	For null hypothesis testing, the test statistic (e.g. F, t, r) with confidence intervals, effect sizes, degrees of freedom and P value noted<br>*Give P values as exact values whenever suitable.*
☒	☐	For Bayesian analysis, information on the choice of priors and Markov chain Monte Carlo settings
☒	☐	For hierarchical and complex designs, identification of the appropriate level for tests and full reporting of outcomes
☒	☐	Estimates of effect sizes (e.g. Cohen's d, Pearson's r), indicating how they were calculated

Our web collection on statistics for biologists contains articles on many of the points above.

Software and code

Policy information about availability of computer code

Data collection	For qRT-qPCR data collection, QIAquant 96 system Software was used. For RNA-seq, the quality of the mRNA-seq libraries was determined using the Agilent Technologies 2100 bioanalyzerthe. For high-throughput SNP genotyping, the Affymetrix SNP chip was used. For the whole-genome resequencing, Illumina HiSeq4000 platform was used. For the observation of stained pollen grains, AxioCam MRc light microscope (Zeiss) was used. For obtaining fluorescence signals, a laser scanning confocal microscope (ZEISS LSM880 or SP8 STED) was used. For collecting visualized western blot signals, a UVP ChemStudio PLUS (Analytik Jena) was used. For in vitro enzymatic analysis, a spectrophotometer (Thermo Scientific, Varioskan LUX) was used.
Data analysis	Data were analyzed by microsoft EXCEL 2013 to perform two-tailed Student's t-test. GraphPad Prism 8.0 version software was used for the multiple comparison test. To retrieve the orthologs of the EAF6 genes, a BLAST approach (v. 2.2.28) was used. All identified EAF6 genes were included in an all-versus-all alignment using MAFFT (v. 7.158b) with the default parameter settings. A maximum likelihood phylogenetic tree was reconstructed using IQ-TREE (v.1.6.3) with 1,000 bootstrap replicates. The synonymous substitution ratio (Ks) of the gene pairs was calculated using ParaAT2.0 software. For transcript quantification, transcripts per million (TPM) values were determined using the Salmon method to normalize the gene expression level in the RNA pools. Transposons and tandem repeats from L. perrieri, O. officinalis, and O. punctata were annotated in detail with the Extensive de novo TE Annotator (EDTA, version1.9.6) using the curated TE library (rice 6.9.5.liban) with the parameters "-overwrite 0 -sensitive 1 -anno 1 -evaluate 0". MCScan software (Python version) was used to scan multiple genomes. For the population genetic analysis, VCFtools was used to calculate the nucleotide diversity (π); BEDTools was used to extract the DNA sequence and the sequences were aligned using MAFFT software; The LD block was displayed using LDBlockShow software. For the introgression analysis, the RectChr softwar was used.<br>For RNA-seq data analysis, RNA-seq reads were aligned to the rice reference genome (MSU-7.0) (http://rice.uga.edu/index.shtml) using Hisat2 (v2.05). Transcription levels of tested genes were quantified by FPKM (fragments per kilobase of exon per million mapped reads). The criteria of fold-change ≥2 and q-value ≤0.05 were established to identify the differentially expressed genes.

For ChIP-seq analysis, the sequence reads were aligned to the rice reference genome (MSU-7.0) (http://rice.uga.edu/index.shtml) and the uniquely mapped reads used for peak calling were analyzed using MACS (v 2.1.4) peak caller software. Motif analysis was conducted using HOMER findMotifsGenome.pl tool and the peaks were annotated using the annotatePeak function in ChIPseeker (v1.26.2). Analyses of the differences between the two samples were performed using the ChIPDiff (2.3.4)program, and the detailed read count data was loaded into the IGV (v2.8.9) genome browser for visualization.
For the genome-wide linkage disequilibrium (LD) analysis, we used a standard one-degree-of-freedom allelic chi-square in Plink(v1.90) with simple 'shell' command.

For manuscripts utilizing custom algorithms or software that are central to the research but not yet described in published literature, software must be made available to editors and reviewers. We strongly encourage code deposition in a community repository (e.g. GitHub). See the Nature Portfolio guidelines for submitting code & software for further information.

Data

Policy information about availability of data

All manuscripts must include a data availability statement. This statement should provide the following information, where applicable:

- Accession codes, unique identifiers, or web links for publicly available datasets
- A description of any restrictions on data availability
- For clinical datasets or third party data, please ensure that the statement adheres to our policy

The query of gene fragment was searched by a web-based tool (https://ricerc.sicau.edu.cn/RiceRC/tools/blastBefore?seq=); The genomes of Zea mays, Hordeum vulgare (Version TRITEX), Leersia perrieri, Oryza brachyantha (FF genome), Oryza punctata (BB genome), and Oryza barthii were all retrieved from ftp://ftp.gramene.org/pub/gramene/release-65/; The genomes of Zizania latifolia and Oryza officinalis (CC genome) were downloaded from the NCBI (GenBank accessions GCA_000418225.1 and GCA_008326285.1, respectively). The genome of Oryza granulata (GG genome) was obtained from the National Genomics Data Center under accession number GWHAAEL00000000. Publicly available data for the genomes of 20 African rice accessions from the rice super pan-genome were obtained from the Genome Warehouse (GWH) (http://bigd.big.ac.cn/gwh/) under PRJCA004295. Data fetching and aggregate of RNA sequencing results from 33 rice varieties were downloaded from the in-house RNA-seq pipeline (BioProject number: PRJCA002103), National Genomics Data Center (http://bigd.big.ac.cn/). The whole-genome repetitive sequence analysis applied to all 33 rice cultivars is accessible in the 'Rice Resource Center' (https://ricerc.sicau.edu.cn/RiceRC/riceInfo/browse).
The The RNA-seq data have been deposited at the National Genomics Data Center under accession number (ID: CRA014308 [https://bigd.big.ac.cn/gsa/browse/CRA014308]).
The The ChIP-seq data have been deposited at the National Genomics Data Center under accession number (ID: CRA014315 [https://bigd.big.ac.cn/gsa/browse/CRA014315])

Research involving human participants, their data, or biological material

Policy information about studies with human participants or human data. See also policy information about sex, gender (identity/presentation), and sexual orientation and race, ethnicity and racism.

Reporting on sex and gender	N/A
Reporting on race, ethnicity, or other socially relevant groupings	N/A
Population characteristics	N/A
Recruitment	N/A
Ethics oversight	N/A

Note that full information on the approval of the study protocol must also be provided in the manuscript.

Field-specific reporting

Please select the one below that is the best fit for your research. If you are not sure, read the appropriate sections before making your selection.

☒ Life sciences ☐ Behavioural & social sciences ☐ Ecological, evolutionary & environmental sciences

For a reference copy of the document with all sections, see nature.com/documents/nr-reporting-summary-flat.pdf

Life sciences study design

All studies must disclose on these points even when the disclosure is negative.

Sample size	Sample size was determined based on our knowledge acquired from our previous experience and from other similar studies. Sample size was described in each figure legend and performed on a minimum number of 3 independent biological replicates. We believe that our sample size is big enough to make our results convictive.

Data exclusions	No data were excluded from the analyses.
Replication	All experiments were independently and successfully conducted for at least two or three times. And the number of biological replications is indicated in the figure legends.
Randomization	Plants were randomly assigned to normal-conditions for phenotype evaluation and sampling for subsequent analysis.
Blinding	No blinding was used in the study due to all genotypes in transgenic materials were independently labeled. Investigators were not blinded because these kinds of experiments did not genetically need blinding.

Reporting for specific materials, systems and methods

We require information from authors about some types of materials, experimental systems and methods used in many studies. Here, indicate whether each material, system or method listed is relevant to your study. If you are not sure if a list item applies to your research, read the appropriate section before selecting a response.

Materials & experimental systems

n/a	Involved in the study	
☐	☒	Antibodies
☒	☐	Eukaryotic cell lines
☒	☐	Palaeontology and archaeology
☒	☐	Animals and other organisms
☒	☐	Clinical data
☒	☐	Dual use research of concern
☐	☒	Plants

Methods

n/a	Involved in the study	
☐	☒	ChIP-seq
☒	☐	Flow cytometry
☒	☐	MRI-based neuroimaging

Antibodies

Antibodies used	For western blotting experiment, primary antibodies: anti-Histone H4 (Abcam, ab177840, rabbit monoclonal), anti-Histone H4Ac (Millipore, 06-866, rabbit monoclonal), anti-Histone H4K5Ac (Abcam, ab51997, rabbit monoclonal), anti-Histone H4K8Ac (Abcam, ab45166, rabbit monoclonal), anti-Histone H4K16Ac (Abcam, ab109463, rabbit monoclonal), anti-Histone H4K20Ac (Abcam, ab177188, rabbit monoclonal), and anti-Histone H4K77Ac (Abcam, ab241117, rabbit polyclonal), anti-Histone H3Ac (Active Motif, 39040, rabbit polyclonal), anti-Histone H2AK5Ac (Active Motif, 39108, rabbit polyclonal), and anti-Histone H2AK9Ac (Active Motif, 39110, rabbit polyclonal). Secondary antibody: goat-Anti-Rabbit IgG (H+L) (Proteintech, SA00001-2). A dilution of 1:2,000 is used for primary and secondary antibodies. For ChIP-seq and ChIP-qPCR experiments: anti-Histone H4Ac (Millipore, 06-866, rabbit monoclonal) is used, with a dilution of 1:200.
Validation	All antibody used are commercial and validations are based on the datasheet from the manufacturer and can found the detail information in the following list:<br>Anti-Histone H4 (https://www.abcam.cn/products/primary-antibodies/histone-h4-antibody-epr16599-chip-grade-ab177840.html).<br>Anti-Histone H4Ac (https://www.merckmillipore.com/CN/zh/product/Anti-acetyl-Histone-H4-Antibody,MM_NF-06-866?ReferrerURL=https%3A%2F%2Fcn.bing.com%2F&bd=1).<br>Anti-Histone H4K5Ac (https://www.abcam.cn/products/primary-antibodies/histone-h4-acetyl-k5-antibody-ep1000y-chip-grade-ab51997.html).<br>Anti-Histone H4K8Ac (https://www.abcam.cn/products/primary-antibodies/histone-h4-acetyl-k8-antibody-ep1002y-chip-grade-ab45166.html).<br>Anti-Histone H4K16Ac (https://www.abcam.cn/products/primary-antibodies/histone-h4-acetyl-k16-antibody-epr1004-ab109463.html).<br>Anti-Histone H4K20Ac (https://www.abcam.cn/products/primary-antibodies/histone-h4-mono-methyl-k20-antibody-epr16999-chip-grade-ab177188.html).<br>Anti-Histone H4K77Ac (https://www.abcam.cn/products/primary-antibodies/histone-h4-acetyl-k77-antibody-ab241117.html).<br>Anti-Histone H3Ac (https://www.activemotif.com/catalog/details/39139/histone-h3ac-pan-acetyl-antibody-pab-1).<br>Anti-Histone H2AK5Ac (https://www.activemotif.com/catalog/details/39107/histone-h2a-acetyl-lys5-antibody-pab).<br>Anti-Histone H2AK9Ac (https://www.activemotif.com/catalog/details/39109/histone-h2a-acetyl-lys9-antibody-pab).

Dual use research of concern

Policy information about dual use research of concern

Hazards

Could the accidental, deliberate or reckless misuse of agents or technologies generated in the work, or the application of information presented in the manuscript, pose a threat to:

No	Yes	
☒	☐	Public health
☒	☐	National security
☒	☐	Crops and/or livestock
☒	☐	Ecosystems
☒	☐	Any other significant area

Experiments of concern

Does the work involve any of these experiments of concern:

No	Yes	
☒	☐	Demonstrate how to render a vaccine ineffective
☒	☐	Confer resistance to therapeutically useful antibiotics or antiviral agents
☒	☐	Enhance the virulence of a pathogen or render a nonpathogen virulent
☒	☐	Increase transmissibility of a pathogen
☒	☐	Alter the host range of a pathogen
☒	☐	Enable evasion of diagnostic/detection modalities
☒	☐	Enable the weaponization of a biological agent or toxin
☒	☐	Any other potentially harmful combination of experiments and agents

Plants

Seed stocks	Rice varities: 'Wuyunjing7' (WYJ7; O.sativa L. ssp. japonica) and CG14 (O.glaberrima Steud.) were used to generate chromosome segment substitution (CSSL) library. The CSSL line, SG178 was used for gene mapping. All the neal isolation lines (NILs) used in this study were generated by using the WYJ7 and SG178.
Novel plant genotypes	N/A
Authentication	PCR based genotyping was used to validate genotypes.

ChIP-seq

Data deposition

☒ Confirm that both raw and final processed data have been deposited in a public database such as GEO.

☐ Confirm that you have deposited or provided access to graph files (e.g. BED files) for the called peaks.

Data access links<br>*May remain private before publication.*	The The ChIP-seq data have been deposited at the National Genomics Data Center under accession number (ID: CRA014315 [https://bigd.big.ac.cn/gsa/browse/CRA014315])
Files in database submission	CRR1006877_f1.fastq.gz<br>CRR1006877_r2.fastq.gz<br>CRR1006878_f1.fastq.gz<br>CRR1006878_r2.fastq.gz<br>CRR1006879_f1.fastq.gz<br>CRR1006879_r2.fastq.gz<br>CRR1006880_f1.fastq.gz<br>CRR1006880_r2.fastq.gz
Genome browser session<br>(e.g. UCSC)	http://rice.uga.edu/cgi-bin/gbrowse/rice/?name=LOC_Os01g13250

Methodology

Replicates	Each ChIP-seq sample with at least three plants but without biological replicate.
Sequencing depth	Each experiment sequence 20M reads, paired-end, length of reads is 75bp.
Antibodies	Anti-Histone H4Ac (Millipore, 06-866, rabbit monoclonal, Lot: 3776112)
Peak calling parameters	MACS2 (version 2.1.4) was used to callpeak with cutoff qvalue < 0.05 by following parameters: callpeak -nomodel -extsize 150 -gsize 4.6,458,000.
Data quality	Raw data (raw reads) of fastq format were firstly processed through in-house perl scripts. In this step, clean data (clean reads) were obtained by removing reads containing adapter, reads containing ploy-N and low quality reads from raw data. At the same time, Q20, Q30 and GC content the clean data were calculated. All the downstream analyses were based on the clean data with high quality.
Software	ChIP-seq data were mapped to rice reference genome (MSU-7.0) (http://rice.uga.edu/index.shtml) by BWA program.<br>Peak calling were analyzed using MACS (v 2.1.4) peak caller software.<br>Motif analysis was conducted using HOMER findMotifsGenome.pl tool and the peaks were annotated using the annotatePeak function in ChIPseeker (v1.26.2).<br>Analyses of the differences between the two samples were performed using the ChIPDiff (2.3.4) program, and the detailed read count data was loaded into the IGV (v2.8.9) genome browser for visualization.
